# Rectal cancer in old age –is it appropriately managed? Evidence from population-based analysis of routine data across the English national health service

**DOI:** 10.1016/j.ejso.2019.01.005

**Published:** 2019-07

**Authors:** Rebecca J. Birch, John C. Taylor, Amy Downing, Katie Spencer, Paul J. Finan, Riccardo A. Audisio, Christopher M. Carrigan, Peter J. Selby, Eva J.A. Morris

**Affiliations:** a Cancer Epidemiology Group, Leeds Institute for Data Analytics, Worsley Building, University of Leeds, LS2 9NL, UK; b Leeds Cancer Centre, Bexley Wing, St James's University Teaching Hospital, Leeds, LS9 7TF, UK; c Department of Surgery, Sahlgrenska University Hospital, Goteborg, Sweden; d Leeds Institute of Cancer and Pathology, St James's University Hospital, Leeds, LS9 7TF, UK

**Keywords:** Colorectal, Cancer, Age, Inequalities, Rectal

## Abstract

**Background:**

There is significant debate as to where to draw the line between undertreating older rectal cancer patients and minimising treatment risks. This study sought to examine the use of radical rectal cancer treatments and associated outcomes in relation to age across the English NHS.

**Methods:**

Patient, tumour and treatment characteristics for all patients diagnosed with a first primary rectal cancer in England between 1st April 2009 and 31st December 2014 were obtained from the CORECT-R data repository. Descriptive analyses and adjusted logistic regression models were undertaken to examine any association between age and the use of major resection and post-surgical outcomes. Funnel plots were used to show variation in adjusted rates of major resection.

**Results:**

The proportion of patients who underwent a major surgical resection fell from 66.5% to 31.7%, amongst those aged <70 and aged ≥80 respectively. After adjustment, 30-day post-operative mortality, failure to rescue and prolonged length of stay were significantly higher among the oldest group when compared to the youngest. Patient reported outcomes were not significantly worse amongst older patients. Significant variation was observed in adjusted surgical resection rates in the oldest patients between NHS Trusts. The probability of death due to cancer was comparable across all age groups.

**Conclusions:**

Older patients who are selected for surgery have good outcomes, often comparable to their younger counterparts. Significant variation in the treatment of older patients could not be explained by differences in measured characteristics and required further investigation.

## 1 Introduction

In 2016 over 9000 rectal cancers were diagnosed in the UK, with 22.5% of these occurring in older patients (aged ≥80) [1]. Increasing life expectancy means that the proportion of the UK population considered very old is predicted to double within the next 25 years [2]. This is likely to result in an increasing number of cancers diagnosed in old and very old people, making their treatment and disease outcomes an important focus for policy makers. The English National Health Service (NHS) is pushing to attain world class cancer outcomes [3] and, to achieve this, it is vital that this large population group receives the highest standard of care. Exactly what constitutes gold standard care for older people is, however, controversial. For example, currently there is major concern about the under-treatment of older people. This has arisen from a growing number of observational studies that have reported lower treatment rates with increasing age [[4], [5], [6]]. In rectal cancer, rates of use of many important treatments, including major surgical resection, neoadjuvant radiotherapy and adjuvant chemotherapy, have all been shown to decrease with increasing age [7,8] and this has been hypothesised to be a contributory factor to UK rectal cancer survival rates lagging behind their European counterparts [4].

Others argue, however, that lower rates of treatment in older people is to be expected. Older age is associated with a higher prevalence of poor prognostic factors including more comorbidity [[9], [10], [11]], delayed presentation [12,13], and later stage disease [14]. These associations make the appropriate treatment rate in relation to age difficult to determine and it is vital they are factored into any analysis investigating what constitutes ‘age appropriate’ care. Furthermore, amongst those older people who are treated, outcomes may be worse than those attained by their younger counterparts. In rectal cancer the figures can be quite alarming, with some studies demonstrating significantly higher proportions dying within 30-days of surgery amongst the oldest age groups [[15], [16], [17], [18], [19], [20]]. This has led some to suggest that major resection may not be appropriate amongst older patients when there is the possibility of an alternative treatment [15]. Similarly, other surgical outcomes such as length of stay in hospital, surgical complications [21] and lower stoma reversal rates [22] may all be worse in older people. So, whilst under-treatment may be a problem for older cancer patients, over-treatment could be equally as important if quality of life is as important as its duration [23]. Concerns have also been raised about the importance clinicians place on various patient characteristics related to age. It has been hypothesised that this leads to the treatment of only those older patients with few adverse factors whilst perceptions of increased risk mean that others, who may have benefitted from treatment, are not provided with the opportunity for optimal treatment [4,24]. There is a fine balance, therefore, between selecting individuals for treatment to minimise risk and not under-treating, or denying treatment, to older age groups. More and better evidence is urgently required to help determine optimal management strategies.

Currently, there is a growing availability of routinely collected healthcare data and its analysis can produce the intelligence needed to help inform the controversy of what constitutes age-appropriate rectal cancer treatment. This study aims to use of these data to examine the use of radical rectal cancer treatments (both surgery and neoadjuvant radiotherapy) and their associated outcomes, and assess how these varied across the English NHS. This information is key in ensuring appropriate and informed treatment for older rectal cancer patients, informing and training practitioners, and delivering improvements in patient outcomes.

## 2 Methods

The COloRECTal cancer data Repository (CORECT-R) [25] links National Cancer Registration and Analysis Service (NCRAS) data to additional, routine national datasets (including both Hospital Episode Statistics (HES) [26] and the Radiotherapy Dataset (RTDS) [27]) to create the richest population-based colorectal dataset possible. From this resource, information was obtained from the National Cancer Registration and Analysis Service (NCRAS) [28] dataset on all patients diagnosed with a first primary rectal cancer (ICD10 [29] code C20) in England between 1st April 2009 and 31st December 2014. Information extracted included age, sex, stage of disease at diagnosis, socioeconomic status (based on the income domain of the Index of Multiple Deprivation (IMD) score), route to diagnosis (RTD) [12,30] and survival time. For ease of interpretation, and in line with the various definitions of old age which have been used by others [4,8,31], patients were grouped into the following age groups; <70, 70–79 and ≥ 80 years at the time of diagnosis.

Patient Reported Outcomes Measures (PROMs) data were available for a subset of patients (patients diagnosed with colorectal cancer between 2010 and 2011 who survived between 12 and 36 months from diagnosis) and were linked into the CORECT-R dataset [32]. Responders were categorised. Based on their EQ-5D score, as having ‘perfect’ (no problems reported on EQ-5D) or less than perfect (one or more problems reported) [32]. The Social Difficulties Inventory (SDI) was used to identify those displaying high levels of social distress based on their score. A score of 10 or more has previously been identified as indicating high levels of social distress and so was used in this study with patients being assigned to one of two groups based on their reposes to the 16 items contained in the SD-16 scale; no social distress – score <10, high levels of social distress – score ≥10 [33,34]. For patients who had a stoma an additional item from the PROMs questionnaire was also examined regarding their level of embarrassment caused by their stoma. Patients were assigned to one of three groups based on their answer to this question (group one – not at all embarrassed, group 2 – a little bit or somewhat embarrassed or group 3 – quite a bit or very much embarrassed).

Details of surgical management, including the use and type of major surgical resection and whether it necessitated the formation of a stoma, were obtained from the HES data in CORECT-R and grouped according to standard algorithms [7,16]. Stoma reversal was identified from procedure codes recorded in the 18 months following its creation. Analysis of stoma reversal included only patients who had survived a minimum of 18 months from stoma creation. Information about neoadjuvant radiotherapy received was obtained from the RTDS for patients who had undergone a major surgical resection. Analysis of PROMs was undertaken to examine the relationship between age and less than perfect health and levels of embarrassment related to a stoma for patients who reported that their stoma had not been reversed. Patients were classified as having; no neoadjuvant radiotherapy, short course radiotherapy with immediate surgery (SCRT-I), short course radiotherapy with delayed surgery (SCRT-D) or long course chemo radiotherapy (LCCRT) again according to standard algorithms [35]. A Charlson comorbidity score was obtained for each patient, based on diagnostic codes (excluding cancer) recorded during any admission to hospital in the year preceding diagnosis. The cancer component of the score, cancer diagnoses prior to the colorectal cancer in question, were derived from the registry data.

For patients who underwent a major surgical resection additional variables were derived; Thirty-day post-operative mortality was defined as a death within 30 days of a major surgical resection [16]. Length of stay (LOS) was defined as the time (in days) between major surgical resection and discharge from hospital or death in hospital, whichever occurred first [21]. Prolonged LOS was defined as being a stay of 21 or more days from surgery [21]. A return to theatre was flagged if an individual was reported as having a procedure to manage a surgical complication within 28 days of their major surgical resection. Failure to rescue was defined as a death within 28 days of a major surgical resection which occurred after at least one return to theatre. Emergency readmission was defined as an emergency admission within 30 days of discharge from the surgical inpatient spell.

Further statistical analyses were conducted limited to the oldest patients (≥80). Multilevel binary logistic regression models were used to assess the factors associated with the probability receipt of a major surgical resection. The models were built with patients clustered within NHS Trusts, allowing for differing population demographics and correlated outcomes between trusts. The explanatory variables included were sex, IMD category, year of diagnosis (included as a continuous variable), Charlson comorbidity score, stage of disease and route to diagnosis (emergency or non-emergency). The outcome of interest was major resection, included as a binary outcome. Funnel plots, produced using the Spiegelhalter method [16,36], were used to compare operative rates between Trusts with the adjusted operative rate plotted against the workload.

Adjusted logistic regression models were used to calculate the odds of 30-day post-operative mortality, return to theatre, failure to rescue, emergency readmission, prolonged length of stay, creation of a stoma during an anterior resection and the presence of a stoma at 18 months from creation. Each outcome was modelled separately and adjusted for age, sex, IMD, Charlson score, stage of disease, year of diagnosis and route to diagnosis (emergency or non-emergency). With the exception of the stoma outcomes, models included all patients who had undergone a major surgical resection of their cancer and included adjustment for the type of operation which was performed (anterior resection, abdominoperineal excision, Hartmann's procedure or other). Further models were produced to examine the odds of less than perfect health or high levels of social distress, as reported in the PROMs data, for patients who underwent a major surgical resection.

Crude probabilities of death due to cancer and due to other causes were calculated for each age and surgical group to allow for an assessment of the probability of dying from cancer in the presence of competing risks [37,38]. Calculations were performed in Stata 15.0 using the strs command with the cuminc option.

All statistical analysis was undertaken using Stata 15.0 (State College, Tx, USA).

## 3 Results

In total, 52,922 people were diagnosed with a first primary rectal cancer in England over the study period. Of these, 11,924 (22.4%) were aged ≥80 at the time of diagnosis (Table 1). A greater proportion of older patients had characteristics associated with poor outcomes compared to their younger counterparts. For example 8.7% of patients aged ≥80 had a Charlson score of ≥3 compared to 1.6% of those aged <70 and 21.3% of patients aged ≥80 were diagnosed as an emergency compared to 7.5% of those aged <70 (Table 1).Overall, 30,134 patients (56.9%) received a major surgical resection. The proportion undergoing a major resection decreased with age, falling from 66.5% amongst those aged <70 to 31.7% amongst those aged ≥80 (Table 1).

**Table 1:** Characteristics of the study population.

		<70		70–79		≥80		Total	
		n	%	n	%	n	%	n	%
**Characteristics**									
Sex	Male	16,719	65.8	10,250	65.8	6514	54.6	33,483	63.3
	Female	8704	34.2	5325	34.2	5410	45.4	19,439	36.7
Socioeconomic status	1 - most affluent	5566	21.9	3451	22.2	2633	22.1	11,650	22.0
	2	5662	22.3	3560	22.9	2668	22.4	11,890	22.5
	3	5337	21.0	3211	20.6	2552	21.4	11,100	21.0
	4	4746	18.7	2929	18.8	2282	19.1	9957	18.8
	5 - most deprived	4112	16.2	2424	15.6	1789	15.0	8325	15.7
Charlson comorbidity score	0	21,151	83.2	10,924	70.1	7218	60.5	39,293	74.2
	1	3196	12.6	2968	19.1	2540	21.3	8704	16.4
	2	679	2.7	960	6.2	1126	9.4	2765	5.2
	≥3	397	1.6	723	4.6	1040	8.7	2160	4.1
Stage of disease	I	4837	19.0	3091	19.8	1756	14.7	9684	18.3
	II	3999	15.7	2868	18.4	1948	16.3	8815	16.7
	III	7640	30.1	4093	26.3	2262	19.0	13,995	26.4
	IV	4064	16.0	2374	15.2	1800	15.1	8238	15.6
	Unknown	4883	19.2	3149	20.2	4158	34.9	12,190	23.0
Route to diagnosis	GP referral	6981	27.5	4183	26.9	3303	27.7	14,467	27.3
	TWW	9379	36.9	6758	43.4	4747	39.8	20,884	39.5
	Emergency	1899	7.5	1606	10.3	2542	21.3	6047	11.4
	Other outpatient	1430	5.6	960	6.2	743	6.2	3133	5.9
	Screening	3711	14.6	1313	8.4	44	0.4	5068	9.6
	Inpatient elective	1168	4.6	500	3.2	356	3.0	2024	3.8
	Unknown	855	3.4	255	1.6	189	1.6	1299	2.4
Total		25,423		15,575		11,924		52,922	
**Management**									
Surgical management	Major resection	16,917	66.5	9440	60.6	3777	31.7	30,134	56.9
	Minor resection	2902	11.4	1857	11.9	1551	13.0	6310	11.9
	Palliative procedure	1536	6.0	975	6.3	927	7.8	3438	6.5
	No surgery	4068	16.0	3303	21.2	5669	47.5	13,040	24.6
	Total	25,423		15,575		11,924		52,922	
Operation type	APE	4055	24.0	2324	24.6	880	23.3	7259	24.1
	Anterior resection	10,667	63.1	5591	59.2	1910	50.6	18,168	60.3
	Hartmann's	903	5.3	838	8.9	713	18.9	2454	8.1
	Other	1292	7.6	687	7.3	274	7.3	2253	7.5
	Total	16,917		9440		3777		30,134	
Radiotherapy^a^	None	8164	48.4	5218	55.4	2608	69.5	15,990	53.2
	SCRT-I	1994	11.8	1142	12.1	353	9.3	3489	11.6
	SCRT-D	149	0.9	130	1.4	160	4.2	439	1.5
	LCCRT	5995	35.4	2563	27.2	465	12.3	9023	29.9
	PORT	333	2.0	186	2.0	83	2.2	602	2.0
	ORT	250	1.5	179	1.9	86	2.3	515	1.7
	Total	16,917		9440		3777		30,134	

a SCRT-I – short course radiotherapy with immediate surgery, SCRT-D – short course radiotherapy with delayed surgery, LCCRT – long course chemo radiotherapy, PORT – post-operative radiotherapy, ORT – other radiotherapy.

In patients undergoing major surgical resection, the use of neoadjuvant radiotherapy decreased with age, with 48.4% of those <70 receiving no radiotherapy compared to 69.5% of those aged ≥80. The use of LCCRT fell (35.4%–12.3%) but was not accounted for by a corresponding increase in the use of SCRT-D, which increased from 0.9% to 4.3% between the youngest and oldest patient groups (Table 1). Little variation in the use of postoperative radiotherapy was identified in relation to age group (Table 1).

**Fig. 1:**
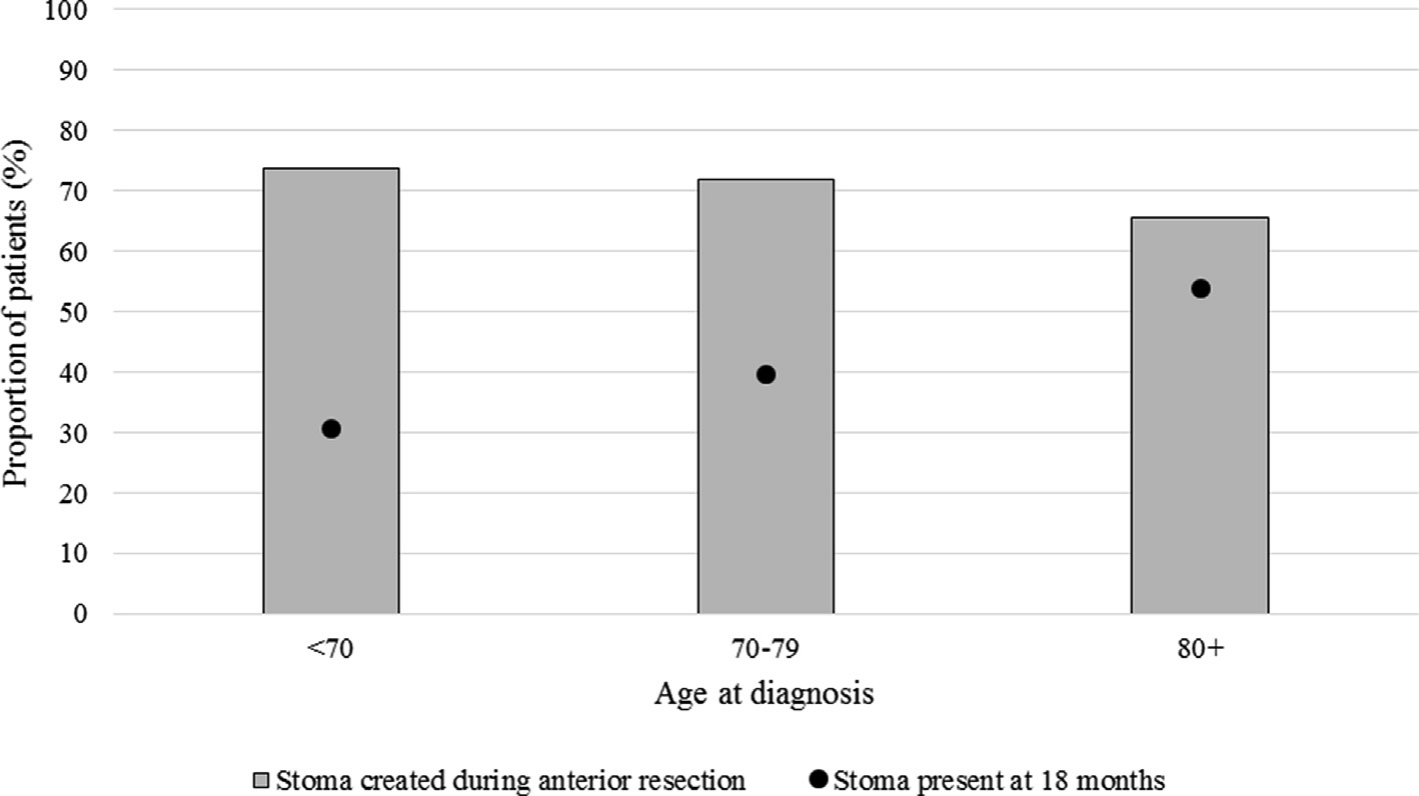
Stoma creation and closure rates for patients who underwent an anterior resection, by age at diagnosis.

**Fig. 2:**
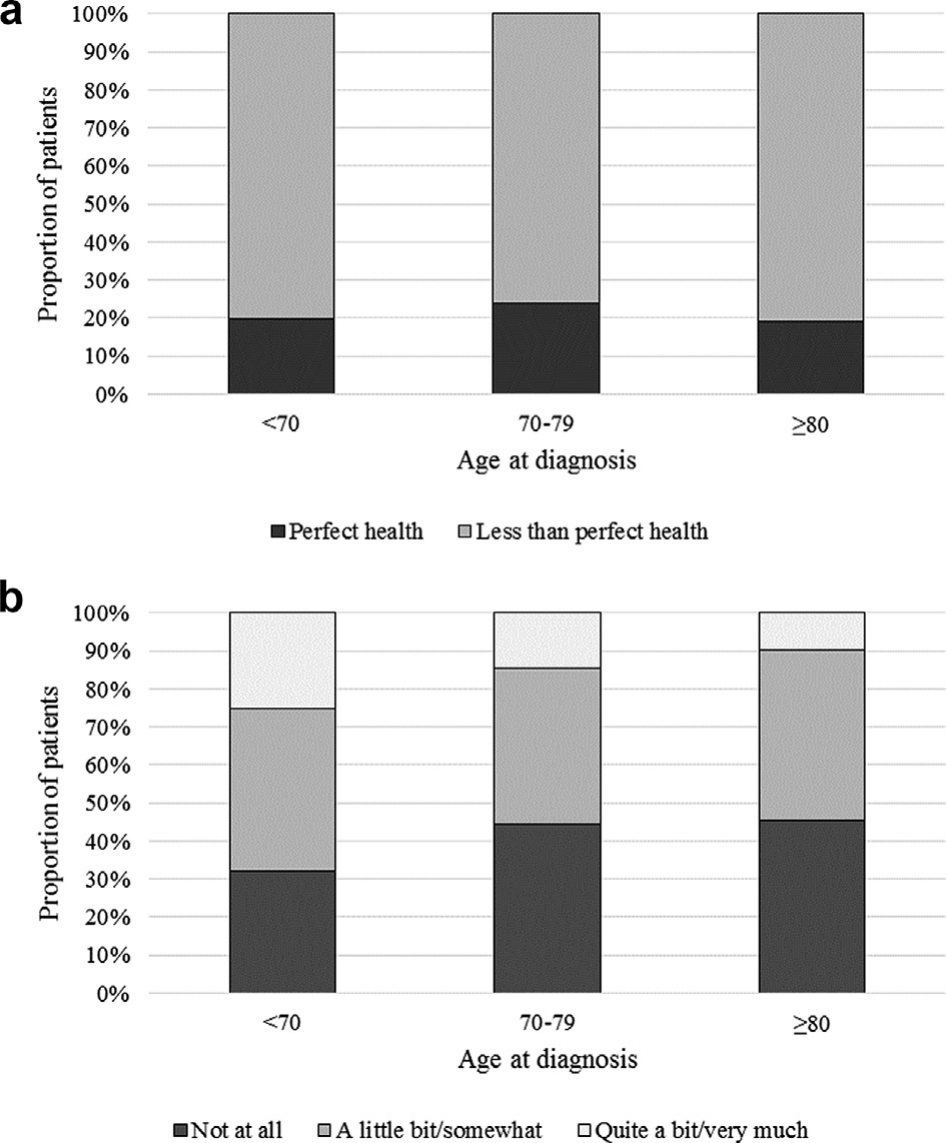
a: Results from PROMs data for patients whose stoma was not reversed - 'Perfect' health. b: Results from PROMs data for patients whose stoma was not reversed - Embarrassed by stoma.

The rate of stoma creation during an anterior resection was similar across all age groups (73.9%, 71.8% and 65.6% respectively). The proportion of patients with a stoma present 18 months from creation, however, increased significantly with age, from 30.6% amongst those aged <70 to 54.0% amongst those aged ≥80 (Fig. 1). The proportion of patients reporting less than ‘perfect’ health in the presence of a permanent stoma did not differ significantly between age groups. Older patients reported lower levels of embarrassment associated with a stoma than their younger counterparts (Fig. 2).

**Fig. 3:**
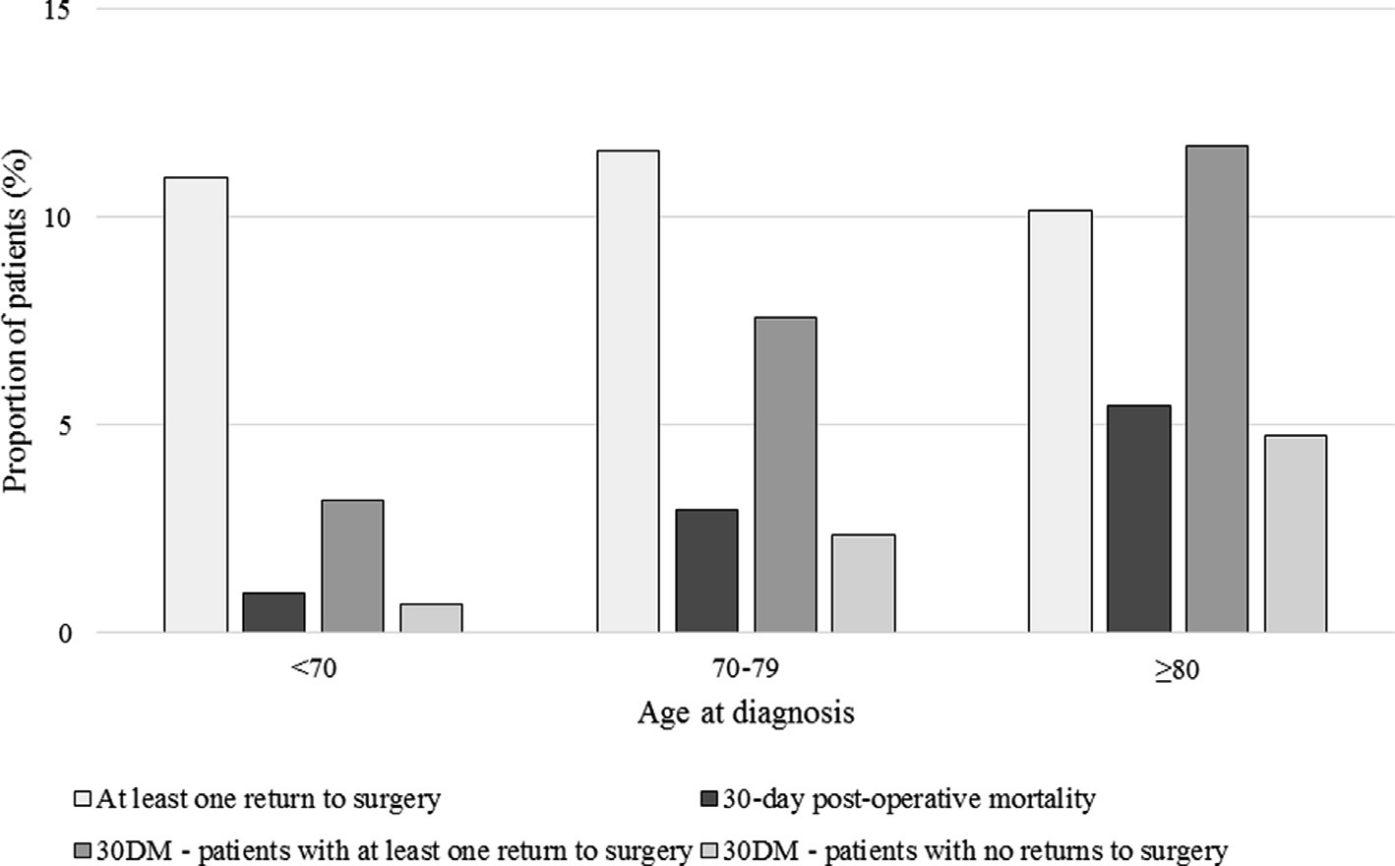
30-day post-operative mortality and return to surgery rates for patients who underwent a major surgical resection, by age at diagnosis.

Deaths within 30 days of a major surgical resection increased with age, from 1.0% to 5.5% in the youngest and oldest groups (Fig. 3). The rate of returns to theatre were relatively consistent between age groups (11.0%, 11.6% and 10.2% respectively) (Fig. 3). Having at least one return to theatre after initial surgery was associated with an increased rate of 30-day post-operative mortality (compared to those who did not return to theatre) and this was consistent across all age groups (Fig. 3). No significant difference was identified in 30-day post-operative mortality between those who received neoadjuvant radiotherapy and those who did not amongst the oldest patients (p > 0.05 results not presented) (Table 2).

**Table 2:** 30-day post-operative mortality and neoadjuvant radiotherapy, by age.

		Status at 30 days from major resection				Total
		Alive		Dead		
		n	%	n	%	
No neoadjuvant radiotherapy	<70	8064	98.8	100	1.2	8164
	70–79	5049	96.8	169	3.2	5218
	≥80	2455	94.1	153	5.9	2608
	Overall	15,588	97.4	422	2.6	16,010
Any neoadjuvant radiotherapy	<70	8080	99.3	58	0.7	8138
	70–79	3736	97.4	99	2.6	3835
	≥80	932	95.3	46	4.7	978
	Overall	12,748	98.4	203	1.6	12,951

**Fig. 4:**
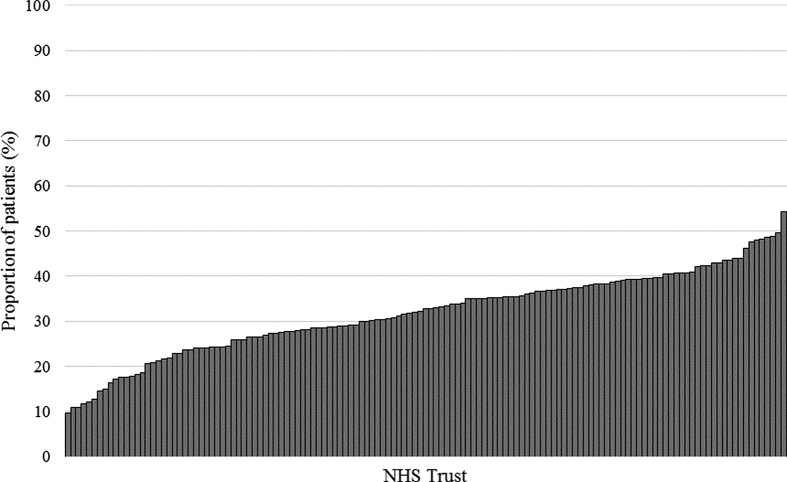

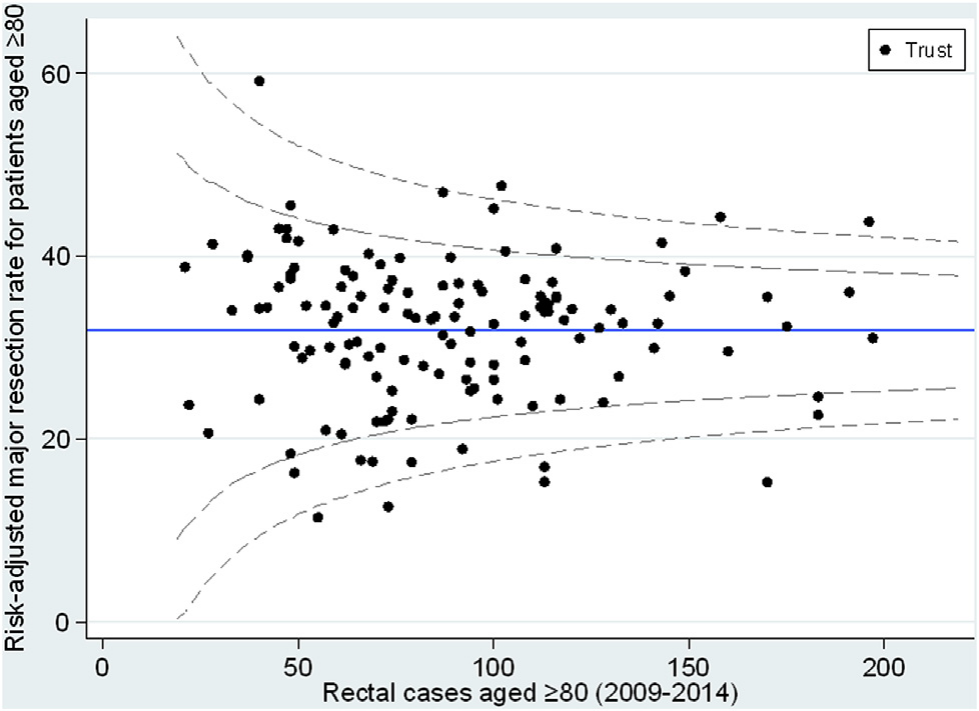
a: Variation in the use of major surgical resection for patients aged ≥80 by NHS Trust. b: Adjusted funnel plot showing rate of resection for patients aged ≥80 by NHS Trust.

Substantial variation in the use of major resection amongst the oldest patients was observed between NHS Trusts in England (rates ranging from 9.7% to 54.2%) (Fig. 4a). After adjustment for casemix factors (sex, IMD category, Charlson comorbidity score, year of diagnosis and stage of disease) significant variation was still observed in operative rates for the oldest patients between NHS Trusts in England (Fig. 4b).

**Fig. 5:**
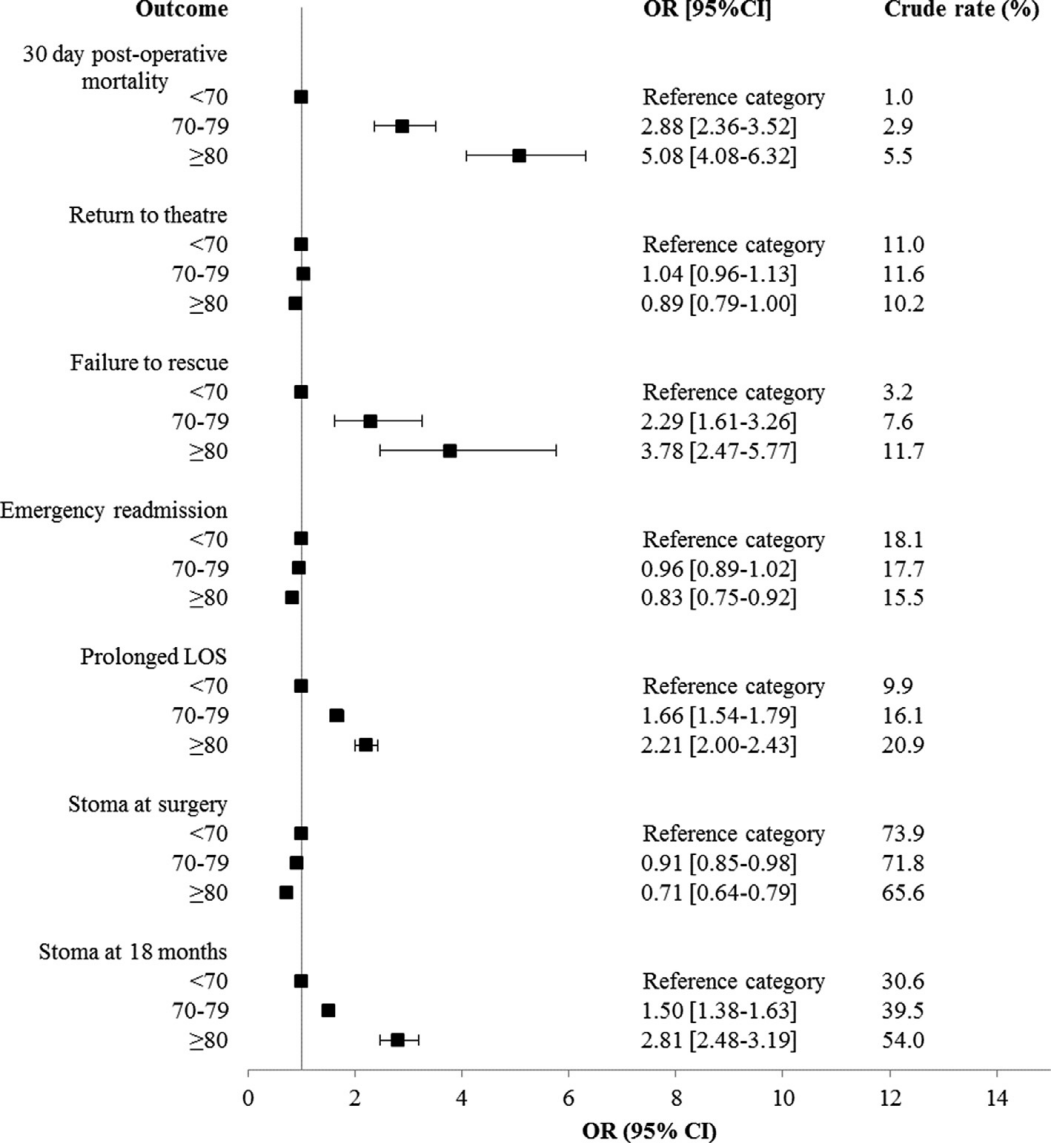
Results of adjusted logistic regression models in relation to age group. Each outcome modelled separately (full results of adjusted models available in Appendix A & C).

**Fig. 6:**
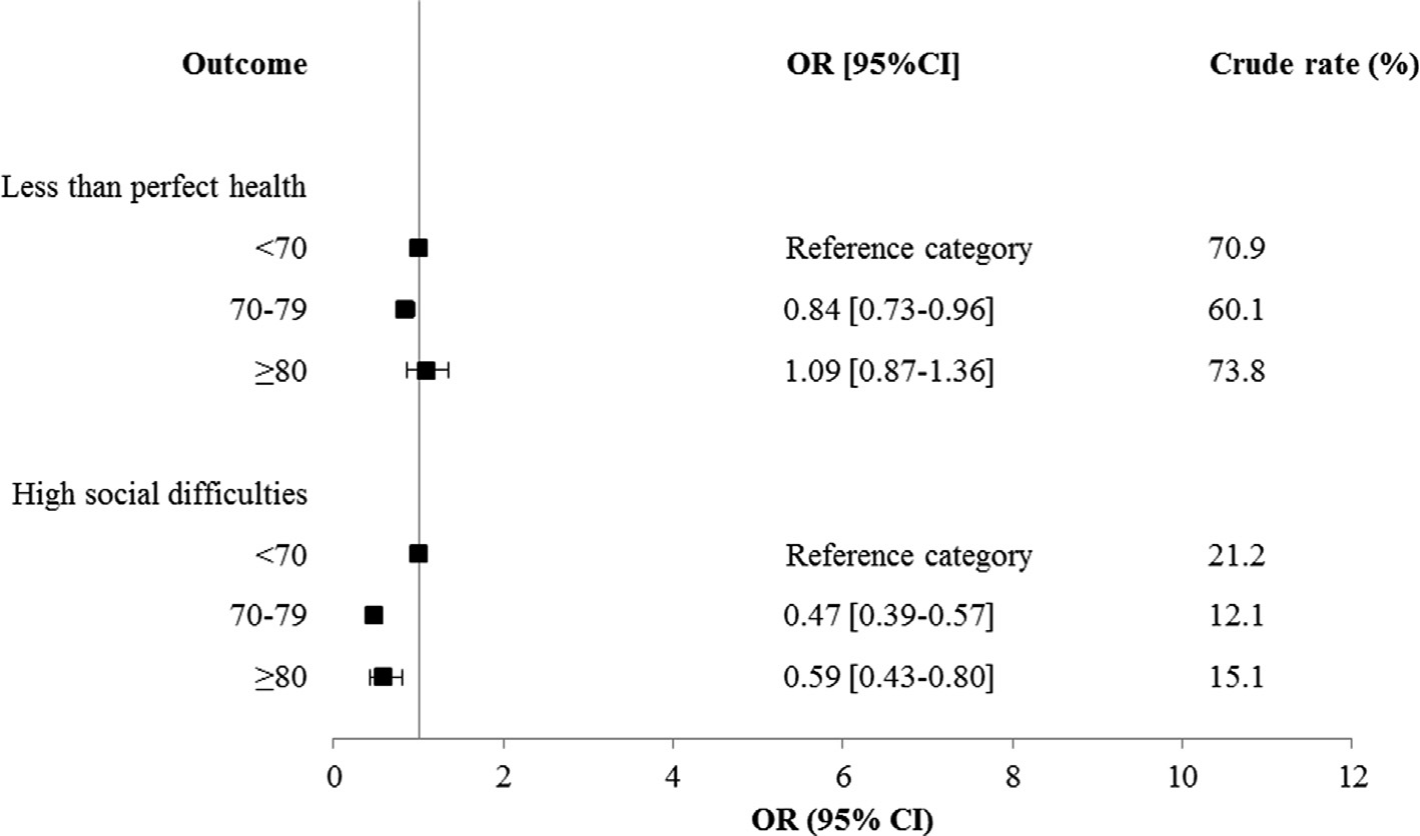
Results of adjusted logistic regression models in relation to age group. Each outcome modelled separately (full results of models available in Appendix D).

Significant variation in 30-day post-operative mortality, failure to rescue, prolonged length of stay and the presence of a stoma at 18 months from creation was identified in relation to age. In adjusted models the odds of all these outcomes was significantly higher amongst the oldest patients compared to the youngest patients. The odds of an emergency readmission or creation of a stoma during an anterior resection fell significantly when comparing the oldest group to the youngest. No significant difference between age groups was identified in relation to the odds of a return to theatre (Fig. 5). No significant difference between age groups was observed in relation to the odds of less than perfect health after a major surgical resection. The odds of high levels social distress were significantly lower amongst individuals aged 70–79 (OR 0.47 95%CI 0.39–0.57) or ≥80 (OR 0.59 95%CI 0.43–0.80) than those aged <70 (Fig. 6).

**Fig. 7:**
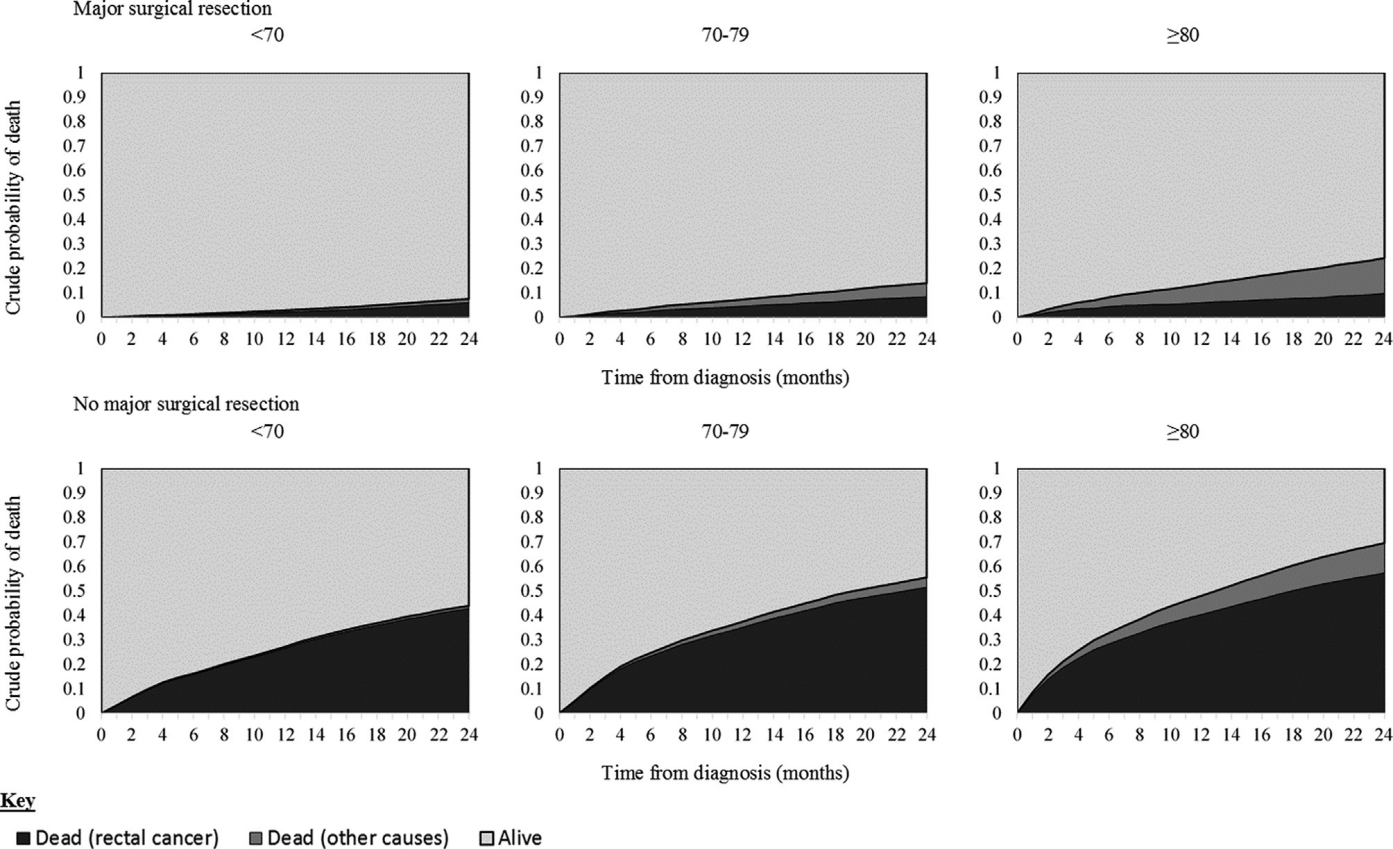
Crude probability of death by age group and surgical management.

Across all age groups the estimated probability of death due to cancer was higher amongst those who did not undergo a major surgical resection. Despite the probability of death due to other causes increasing with age the probability of death due to cancer was comparable between age groups in both treatment categories (major resection and no major resection) (Fig. 7). Within the oldest group (≥80) who underwent a major resection 9.8% of patients were predicted to die, due to cancer, within 24 months of diagnosis, compared to 5.9% of those aged < 70. In contrast deaths due to other causes rose significantly with 14.5% of all patients aged ≥80 who underwent a major resection dying due to other causes within 24 months of diagnosis, compared to only 1.6% of those aged <70.

## 4 Discussion

This comprehensive analysis of national, population-based datasets of rectal cancers managed within the English NHS provides valuable information to inform the management of older people. It has shown that, although the risks of radical treatment are greater in older people their outcomes can be comparable to those obtained in younger age groups. It also demonstrated, however, that older people were significantly less likely to receive potentially curative treatments than their younger counterparts and, although contributory to it, this variation is not fully explained by differences in the distribution of important prognostic factors related to age (and that can be measured in routine data) such as increasing Charlson comorbidity score or stage of disease. Significant variation was also apparent in how different NHS hospital Trusts actively managed older patients and this, again, was not explained by casemix differences. Minimising these variations is vital to improve rectal cancer care and outcomes in the NHS. There were, however, other relevant factors, such as frailty and patient choice, which were not quantified in the available data and which may contribute to the variation observed.

These data should help both patients and the multidisciplinary teams who manage them to make informed decisions about treatment. For example, when surgeons discuss treatment options with older rectal cancer patients they can refer to this very large ‘real world’ study of the results achieved in the English NHS when they are considering the roll of radical surgery to inform patients of the risks and benefits of treatment.

The oldest patients were less likely than their younger counterparts to undergo a major surgical resection or neoadjuvant radiotherapy as a treatment for rectal cancer. Our data shows that some of this reduction is associated with clinical characteristics such as comorbidity and appears to reflect appropriate case selection in many cases. Our data do hint that patients, clinicians, or both may give a greater weight to comorbidity in older patients when making decisions. The measure of comorbidity used in this study is, however, blunt, identifying comorbid conditions through inpatient admission records. A more robust measure may offer greater insight into specific conditions or health needs that may contribute to this. Under some circumstances avoiding surgery in older people may be appropriate if clinicians are concerned about risk factors that are not identifiable from routine data. If, however, the variation seen is a result of an inappropriate emphasis on chronological age then action must be taken to ensure no-one is denied potentially curative treatment.

Amongst the oldest patients it is of note that the probability of death due to cancer amongst those who underwent a major surgical resection for their rectal tumour was lower than the probability of death due to other causes and was, in fact, comparable to the probabilities observed amongst the youngest patients. Supporting the suggestion that the oldest patients can achieve outcomes in line with those of their younger counterparts. For those who did undergo a major resection of their tumour, older patients were no more likely to report less than perfect health than their younger counterparts. In contrast, older patients were in fact less likely to report high levels of social distress than younger patients following major surgery for their rectal cancer. Older patients often rate quality of life as being more important to them than higher duration of survival [23,39]. The findings of this study demonstrate that there does not appear to be a direct relationship between increasing age, intensity of treatment, and poor quality of life, suggesting that this should not be a reason not to offer treatment.

Deaths within 30-days of surgery increased with age, but even in the oldest patients failed to reach the rate cited by others as an unacceptable risk [15,20]. While returns to surgery were similar across age groups, deaths within 30-days amongst patients who had returned to surgery increased with age to a greater extent than was seen amongst those with no returns to surgery. The reasons for this are unclear and require further investigation, but may reflect the reduced ability of the oldest patients to cope with the physiological insult necessitating the return to theatre.

Notably individuals receiving neoadjuvant radiotherapy did not have higher rates of post-operative mortality, irrespective of age. The concern that the use of radiotherapy might worsen post-operative outcomes disproportionately in the older age group is not justified by these data. There is undoubtedly a significant element of case selection currently which requires further investigation, as does the finding that reduced use of LCCRT in the older age group is not compensated for by increased use of SCRT-D.

Whilst surgery remains the gold standard of care, there is an increasing recognition of the potential role of chemoradiotherapy or radiotherapy alone as a curative treatment, particularly in those considered to be at high operative risk. This cannot be addressed with the available data. This alternative treatment is likely only to be used in a very small number of cases, with access to contact brachytherapy in particular varying markedly across the country. On this basis it seems unlikely that non-surgical approaches are significantly impacting upon the overall results seen here, although these may explain some specific areas of the geographical variation.

This study shows stoma reversal rates are lower in older patients. Studies have reported that stomas in older patients are not associated with the reduction in quality of life which is often seen amongst younger patients, and some studies suggest stomas may be associated with an improved quality of life amongst older patients [[40], [41], [42]], meaning that non reversal is not necessarily a negative outcome. This is supported by findings from the PROMs data used in this study which found no difference in reported ‘perfect’ health between age groups. Further work is needed to determine whether low reversal rates are due to patient choice, surgeon choice or reflect advanced stage of presentation.

Further casemix information is needed in order to truly understand the variation observed between Trusts. NBOCA collects information about the reason why patients did not undergo a major resection, including too little cancer, too much cancer, high levels of frailty and other/unknown reasons. An assessment of any variation in these factors between Trusts could provide important additional information regarding the selection process [43].

A significant limitation of this study is that it includes no measure of patient choice. Some have suggested that older patients may be more likely to refuse treatment as they often rate quality of life over quantity [23,44]. However, a recent study of 1500 patients found that older patients were no more likely to refuse treatment which is offered than their younger counterparts [45]. This finding suggests that treatment refusal is unlikely to account for the fall in treatment rates observed, but further work to assess patient choice and shared decision making is key to understanding differences in observed treatment rates.

## 5 Conclusions

This study provides the first comprehensive, population-based, description of the initial management of rectal cancer patients in the English NHS. It demonstrates that older patients undergoing treatment for rectal cancer have comparable outcomes to their younger counterparts. It identifies important shortfalls in uptake of major treatments and suggests explanations. Understanding these issues should inform policy and service planning and practice within MDTs. Greater national consistency should improve outcomes, which remain relatively poor in older patients.

## Declarations

### Ethics approval and consent to participate

This study was approved by Multicentre Research and Ethics Committee (MREC) (Newcastle and North Tyneside Research Ethics Committee ref 14/NE/0007 and East of Scotland Research Ethics Committee ref 08/S0501/66).

### Availability of data and material

The data used for this study are available from the National Cancer Registration and Analysis Service via the PHE Office for Data Release, subject to relevant approvals.

### Role of the funding source

This work is supported by Cancer Research UK (grants C23434/A23706 and C34080/A16438). Cancer Research UK had no role in the study design, collection, analysis or interpretation of the data, the writing of the manuscript or the decision to submit for publication.

### Authors contributions

All authors contributed to the conception and design of the study, the interpretation of the results and the writing of the manuscript. RJB, JCT, AD and EJAM undertook the analysis of the data. All authors commented on and approved the final manuscript.

## Appendix A Supplementary data

Appendix A: Adjusted logistic regression model results for patients who have undergone a major surgical resection (each outcome modelled separately).

Appendix B: Univariate logistic regression model results for patients who have undergone a major surgical resection (each outcome modelled separately).

Appendix C: Results from multivariate and univariate logistic regression models for patients who underwent an anterior resection (each outcome modelled separately).

Appendix D: Results from multivariate and univariate logistic regression models using the PROMs data (each outcome modelled separately).
