# Supplementary material for: Rectal cancer in old age –is it appropriately managed? Evidence from population-based analysis of routine data across the English national health service
Source: Eur J Surg Oncol. 2019 Jul;45(7):1196–204. doi: 10.1016/j.ejso.2019.01.005 (PMC6602152; doi:10.1016/j.ejso.2019.01.005)
Supplement: Multimedia component 1 [file mmc1.docx]

|  |  | 30-day post-operative mortality | | Return to theatre | | Failure to rescue | | Emergency readmission | | Prolonged length of stay | |
| --- | --- | --- | --- | --- | --- | --- | --- | --- | --- | --- | --- |
|  |  | OR | 95%CI | OR | 95%CI | OR | 95%CI | OR | 95%CI | OR | 95%CI |
| Age group | <70 | 1.00 |  | 1.00 |  | 1.00 |  | 1.00 |  | 1.00 |  |
|  | 70-79 | 2.88 | 2.36-3.52 | 1.04 | 0.96-1.13 | 2.29 | 1.61-3.26 | 0.96 | 0.89-1.02 | 1.66 | 1.54-1.79 |
|  | ≥80 | 5.08 | 4.08-6.32 | 0.89 | 0.79-1.00 | 3.78 | 2.47-5.77 | 0.83 | 0.75-0.92 | 2.21 | 2.00-2.43 |
| Sex | Male | 1.00 |  | 1.00 |  | 1.00 |  | 1.00 |  | 1.00 |  |
|  | Female | 0.54 | 0.45-0.65 | 0.72 | 0.66-0.78 | 0.81 | 0.57-1.15 | 0.82 | 0.77-0.88 | 0.74 | 0.69-0.80 |
| Socioeconomic status (IMD) | 1 – most affluent | 1.00 |  | 1.00 |  | 1.00 |  | 1.00 |  | 1.00 |  |
|  | 2 | 1.00 | 0.79-1.28 | 1.00 | 0.90-1.12 | 0.86 | 0.54-1.39 | 1.02 | 0.93-1.12 | 1.04 | 0.94-1.15 |
|  | 3 | 0.99 | 0.77-1.27 | 1.04 | 0.93-1.16 | 1.05 | 0.66-1.67 | 1.09 | 0.99-1.19 | 1.08 | 0.97-1.20 |
|  | 4 | 1.14 | 0.88-1.46 | 1.11 | 0.99-1.24 | 0.96 | 0.60-1.56 | 1.19 | 1.09-1.31 | 1.22 | 1.09-1.35 |
|  | 5 – most deprived | 1.21 | 0.93-1.58 | 1.16 | 1.03-1.31 | 1.30 | 0.80-2.11 | 1.18 | 1.07-1.31 | 1.47 | 1.32-1.64 |
| Charlson comorbidity score | 0 | 1.00 |  | 1.00 |  | 1.00 |  | 1.00 |  | 1.00 |  |
|  | 1 | 1.28 | 1.04-1.58 | 1.09 | 0.98-1.21 | 1.01 | 0.66-1.55 | 1.19 | 1.10-1.30 | 1.33 | 1.21-1.45 |
|  | 2 | 1.82 | 1.34-2.47 | 1.42 | 1.19-1.70 | 1.69 | 0.96-2.95 | 1.35 | 1.16-1.57 | 1.95 | 1.68-2.27 |
|  | ≥3 | 3.10 | 2.25-4.29 | 1.34 | 1.06-1.71 | 4.32 | 2.41-7.75 | 1.33 | 1.08-1.64 | 1.81 | 1.48-2.21 |
| Stage of disease | I | 1.00 |  | 1.00 |  | 1.00 |  | 1.00 |  | 1.00 |  |
|  | II | 1.40 | 1.09-1.79 | 1.13 | 1.01-1.26 | 1.82 | 1.11-2.98 | 1.07 | 0.98-1.17 | 1.17 | 1.06-1.30 |
|  | III | 1.35 | 1.06-1.72 | 1.22 | 1.11-1.35 | 1.56 | 0.97-2.51 | 1.14 | 1.05-1.23 | 1.17 | 1.06-1.29 |
|  | IV | 1.60 | 1.12-2.28 | 0.99 | 0.83-1.18 | 1.29 | 0.58-2.84 | 1.10 | 0.96-1.26 | 1.15 | 0.98-1.35 |
|  | Unknown | 1.32 | 0.97-1.79 | 1.08 | 0.94-1.23 | 1.40 | 0.78-2.61 | 1.14 | 1.02-1.27 | 1.17 | 1.03-1.33 |
| Year of diagnosis | | 0.88 | 0.84-0.93 | 0.97 | 0.95-0.99 | 0.93 | 0.84-1.02 | 0.99 | 0.98-1.01 | 0.93 | 0.91-0.95 |
| Route to diagnosis | Non-emergency | 1.00 |  | 1.00 |  | 1.00 |  | 1.00 |  | 1.00 |  |
|  | Emergency | 2.72 | 2.18-3.40 | 1.26 | 1.09-1.47 | 2.14 | 1.35-3.40 | 1.04 | 0.91-1.19 | 1.77 | 1.57-2.01 |
| Operation | Abdominoperineal excision | 1.00 |  | 1.00 |  | 1.00 |  | 1.00 |  | 1.00 |  |
|  | Anterior resection | 1.26 | 1.01-1.57 | 0.85 | 0.78-0.92 | 1.99 | 1.30-3.05 | 1.01 | 0.94-1.08 | 0.81 | 0.74-0.87 |
|  | Hartmann’s procedure | 2.08 | 1.58-2.74 | 1.01 | 0.87-1.16 | 2.67 | 1.56-4.57 | 0.96 | 0.84-1.08 | 1.15 | 1.02-1.31 |
|  | Other | 2.65 | 2.00-3.52 | 0.88 | 0.76-1.03 | 1.68 | 0.86-3.28 | 0.99 | 0.88-1.12 | 1.10 | 0.96-1.26 |
