# Supplementary material for: Rectal cancer in old age –is it appropriately managed? Evidence from population-based analysis of routine data across the English national health service
Source: Eur J Surg Oncol. 2019 Jul;45(7):1196–204. doi: 10.1016/j.ejso.2019.01.005 (PMC6602152; doi:10.1016/j.ejso.2019.01.005)
Supplement: Multimedia component 2 [file mmc2.docx]

|  |  | 30-day post-operative mortality | | Return to theatre | | Failure to rescue | | Emergency readmission | | Prolonged length of stay | |
| --- | --- | --- | --- | --- | --- | --- | --- | --- | --- | --- | --- |
|  |  | OR | 95%CI | OR | 95%CI | OR | 95%CI | OR | 95%CI | OR | 95%CI |
| Age group | <70 | 1.00 |  | 1.00 |  | 1.00 |  | 1.00 |  | 1.00 |  |
|  | 70-79 | 3.16 | 2.60-3.84 | 1.07 | 0.99-1.16 | 2.49 | 1.77-3.51 | 0.97 | 0.91-1.04 | 1.76 | 1.63-1.89 |
|  | ≥80 | 6.00 | 4.87-7.40 | 0.92 | 0.82-1.03 | 4.04 | 2.70-6.06 | 0.83 | 0.75-0.92 | 2.41 | 2.20-2.65 |
| Sex | Male | 1.00 |  | 1.00 |  | 1.00 |  | 1.00 |  | 1.00 |  |
|  | Female | 0.59 | 0.49-0.71 | 0.71 | 0.66-0.77 | 0.85 | 0.60-1.19 | 0.81 | 0.76-0.86 | 0.76 | 0.71-0.82 |
| Socioeconomic status (IMD) | 1 – most affluent | 1.00 |  | 1.00 |  | 1.00 |  | 1.00 |  | 1.00 |  |
|  | 2 | 1.07 | 0.84-1.35 | 1.02 | 0.91-1.34 | 0.89 | 0.56-1.41 | 1.03 | 0.94-1.13 | 1.07 | 0.96-1.18 |
|  | 3 | 1.06 | 0.83-1.36 | 1.06 | 0.95-1.19 | 1.02 | 0.65-1.61 | 1.10 | 1.00-1.20 | 1.11 | 1.00-1.24 |
|  | 4 | 1.24 | 0.97-1.58 | 1.14 | 1.02-1.28 | 1.05 | 0.66-1.67 | 1.22 | 1.11-1.33 | 1.27 | 1.14-1.41 |
|  | 5 – most deprived | 1.37 | 1.07-1.77 | 1.23 | 1.09-1.38 | 1.23 | 0.77-1.97 | 1.23 | 1.11-1.35 | 1.57 | 1.41-1.75 |
| Charlson comorbidity score | 0 | 1.00 |  | 1.00 |  | 1.00 |  | 1.00 |  | 1.00 |  |
|  | 1 | 1.68 | 1.37-2.06 | 1.12 | 1.01-1.24 | 1.22 | 0.81-1.85 | 1.19 | 1.10-1.30 | 1.52 | 1.39-1.66 |
|  | 2 | 3.04 | 2.28-4.07 | 1.50 | 1.26-1.78 | 2.37 | 1.39-4.04 | 1.35 | 1.16-1.56 | 2.52 | 2.18-2.91 |
|  | ≥3 | 5.81 | 4.30-7.85 | 1.42 | 1.12-1.80 | 6.09 | 3.53-10.49 | 1.32 | 1.07-1.62 | 2.50 | 2.06-3.03 |
| Stage of disease | I | 1.00 |  | 1.00 |  | 1.00 |  | 1.00 |  | 1.00 |  |
|  | II | 1.66 | 1.30-2.12 | 1.15 | 1.03-1.28 | 1.95 | 1.21-3.15 | 1.07 | 0.98-1.17 | 1.27 | 1.14-1.40 |
|  | III | 1.32 | 1.04-1.67 | 1.23 | 1.11-1.36 | 1.45 | 0.91-2.31 | 1.14 | 1.05-1.23 | 1.15 | 1.04-1.26 |
|  | IV | 1.73 | 1.23-2.44 | 1.03 | 0.87-1.22 | 1.67 | 0.64-2.90 | 1.11 | 0.97-1.27 | 1.17 | 1.01-1.37 |
|  | Unknown | 1.57 | 1.17-2.10 | 1.15 | 1.01-1.31 | 1.32 | 0.72-2.41 | 1.15 | 1.04-1.28 | 1.30 | 1.15-1.47 |
| Year of diagnosis | | 0.88 | 0.84-0.92 | 0.98 | 0.95-1.00 | 0.92 | 0.84-1.01 | 1.00 | 0.98-1.01 | 0.93 | 0.91-0.95 |
| Route to diagnosis | Non-emergency | 1.00 |  | 1.00 |  | 1.00 |  | 1.00 |  | 1.00 |  |
|  | Emergency | 4.35 | 3.55-5.33 | 1.36 | 1.17-1.57 | 2.65 | 1.74-4.04 | 1.09 | 0.96-1.24 | 2.31 | 2.05-2.60 |
| Operation | Abdominoperineal excision | 1.00 |  | 1.00 |  | 1.00 |  | 1.00 |  | 1.00 |  |
|  | Anterior resection | 1.18 | 0.94-1.47 | 0.84 | 0.77-0.92 | 1.83 | 1.20-2.77 | 1.00 | 0.93-1.07 | 0.78 | 0.72-0.84 |
|  | Hartmann’s procedure | 3.34 | 2.57-4.36 | 1.03 | 0.90-1.18 | 3.44 | 2.04-5.78 | 0.96 | 0.85-1.08 | 1.48 | 1.31-1.67 |
|  | Other | 3.14 | 2.39-4.13 | 0.90 | 0.78-1.05 | 1.88 | 0.99-3.56 | 1.01 | 0.89-1.14 | 1.19 | 1.04-1.35 |
