# Supplementary material for: Rectal cancer in old age –is it appropriately managed? Evidence from population-based analysis of routine data across the English national health service
Source: Eur J Surg Oncol. 2019 Jul;45(7):1196–204. doi: 10.1016/j.ejso.2019.01.005 (PMC6602152; doi:10.1016/j.ejso.2019.01.005)
Supplement: Multimedia component 3 [file mmc3.docx]

|  |  | Adjusted | | | | Unadjusted | | | |
| --- | --- | --- | --- | --- | --- | --- | --- | --- | --- |
|  |  | Stoma creation (patients with an anterior resection) | | Stoma present at 18 months for patients with a stoma created during an anterior resection | | Stoma creation (patients with an anterior resection) | | Stoma present at 18 months for patients with a stoma created during an anterior resection | |
|  |  | OR | 95%CI | OR | 95%CI | OR | 95%CI | OR | 95%CI |
| Age group | <70 | 1.00 |  | 1.00 |  | 1.00 |  | 1.00 |  |
|  | 70-79 | 0.91 | 0.85-0.98 | 1.50 | 1.38-1.63 | 0.90 | 0.84-0.97 | 1.48 | 1.37-1.60 |
|  | ≥80 | 0.71 | 0.64-0.79 | 2.81 | 2.48-3.19 | 0.67 | 0.61-0.75 | 2.66 | 2.36-3.00 |
| Sex | Male | 1.00 |  | 1.00 |  | 1.00 |  | 1.00 |  |
|  | Female | 0.74 | 0.69-0.79 | 0.98 | 0.90-1.06 | 0.73 | 0.69-0.79 | 0.97 | 0.90-1.05 |
| Socioeconomic status (IMD) | 1 – most affluent | 1.00 |  | 1.00 |  | 1.00 |  | 1.00 |  |
|  | 2 | 1.01 | 0.92-1.11 | 1.15 | 1.03-1.28 | 1.01 | 0.92-1.11 | 1.17 | 1.05-1.30 |
|  | 3 | 1.03 | 0.93-1.13 | 1.14 | 1.02-1.28 | 1.03 | 0.94-1.14 | 1.15 | 1.03-1.28 |
|  | 4 | 1.03 | 0.93-1.15 | 1.28 | 1.14-1.44 | 1.03 | 0.93-1.14 | 1.30 | 1.16-1.45 |
|  | 5 – most deprived | 1.00 | 0.89-1.12 | 1.50 | 1.33-1.70 | 1.01 | 0.90-1.12 | 1.55 | 1.37-1.74 |
| Charlson comorbidity score | 0 | 1.00 |  | 1.00 |  | 1.00 |  | 1.00 |  |
|  | 1 | 0.93 | 0.84-1.02 | 1.34 | 1.20-1.49 | 0.92 | 0.83-1.01 | 1.46 | 1.31-1.61 |
|  | 2 | 1.01 | 0.84-1.22 | 1.68 | 1.37-2.06 | 0.99 | 0.82-1.19 | 2.01 | 1.65-2.45 |
|  | ≥3 | 0.88 | 0.68-1.14 | 2.04 | 1.52-2.74 | 0.84 | 0.65-1.08 | 2.43 | 1.83-3.22 |
| Stage of disease | I | 1.00 |  | 1.00 |  | 1.00 |  | 1.00 |  |
|  | II | 0.91 | 0.83-1.00 | 1.44 | 1.29-1.69 | 0.90 | 0.82-0.99 | 1.51 | 1.34-1.69 |
|  | III | 1.10 | 1.01-1.20 | 1.96 | 1.77-2.17 | 1.13 | 1.03-1.23 | 1.86 | 1.68-2.05 |
|  | IV | 0.99 | 0.85-1.16 | 3.69 | 3.11-4.38 | 1.02 | 0.88-1.19 | 3.32 | 2.81-3.93 |
|  | Unknown | 1.38 | 1.21-1.58 | 1.97 | 1.71-2.27 | 1.33 | 1.18-1.52 | 1.79 | 1.57-2.06 |
| Year of diagnosis | | 1.07 | 1.04-1.09 | 1.02 | 1.00-1.04 | 1.06 | 1.04-1.08 | 1.02 | 1.00-1.04 |
| Route to diagnosis | Non-emergency | 1.00 |  | 1.00 |  | 1.00 |  | 1.00 |  |
|  | Emergency | 0.90 | 0.76-1.08 | 1.66 | 1.36-2.02 | 0.85 | 0.72-1.01 | 2.07 | 1.71-2.50 |
