# Supplementary material for: Rectal cancer in old age –is it appropriately managed? Evidence from population-based analysis of routine data across the English national health service
Source: Eur J Surg Oncol. 2019 Jul;45(7):1196–204. doi: 10.1016/j.ejso.2019.01.005 (PMC6602152; doi:10.1016/j.ejso.2019.01.005)
Supplement: Multimedia component 4 [file mmc4.docx]

|  |  | Adjusted | | | | Unadjusted | | | |
| --- | --- | --- | --- | --- | --- | --- | --- | --- | --- |
|  |  | Less than perfect health | | High levels of social distress | | Less than perfect health | | High levels of social distress | |
|  |  | OR | 95%CI | OR | 95%CI | OR | 95%CI | OR | 95%CI |
| Age group | <70 | 1.00 |  | 1.00 |  | 1.00 |  | 1.00 |  |
|  | 70-79 | 0.84 | 0.73-0.96 | 0.47 | 0.39-0.57 | 0.88 | 0.77-1.00 | 0.51 | 0.42-6.2 |
|  | ≥80 | 1.09 | 0.87-1.36 | 0.59 | 0.43-0.80 | 1.16 | 0.94-1.43 | 0.66 | 0.49-0.88 |
| Sex | Male | 1.00 |  | 1.00 |  | 1.00 |  | 1.00 |  |
|  | Female | 1.05 | 0.92-1.19 | 1.07 | 0.91-1.26 | 1.02 | 0.90-1.15 | 1.05 | 0.89-1.23 |
| Socioeconomic status (IMD) | 1 – most affluent | 1.00 |  | 1.00 |  | 1.00 |  | 1.00 |  |
|  | 2 | 1.07 | 0.91-1.27 | 0.98 | 0.77-1.24 | 1.10 | 0.93-1.30 | 1.02 | 0.81-1.29 |
|  | 3 | 1.44 | 1.21-1.72 | 1.35 | 1.07-1.71 | 1.44 | 1.21-1.71 | 1.39 | 1.11-1.75 |
|  | 4 | 1.16 | 0.96-1.40 | 1.42 | 1.11-1.83 | 1.27 | 1.06-1.52 | 1.61 | 1.27-2.04 |
|  | 5 – most deprived | 1.31 | 1.05-1.64 | 1.93 | 1.48-2.53 | 1.49 | 1.20-1.84 | 2.17 | 1.68-2.80 |
| Non cancer Charlson comorbidity score | 0 | 1.00 |  | 1.00 |  | 1.00 |  | 1.00 |  |
|  | 1 | 1.45 | 1.18-1.77 | 1.44 | 1.14-1.81 | 1.46 | 1.20-1.77 | 1.45 | 1.17-1.82 |
|  | 2 | 2.24 | 1.33-3.75 | 1.99 | 1.19-3.32 | 2.31 | 1.39-3.84 | 2.00 | 1.24-3.24 |
|  | ≥3 | 2.19 | 0.96-4.97 | 2.23 | 1.06-4.68 | 2.36 | 1.05-5.31 | 2.16 | 1.06-4.42 |
| Stage of disease | I | 1.00 |  | 1.00 |  | 1.00 |  | 1.00 |  |
|  | II | 1.11 | 0.94-1.32 | 1.37 | 1.07-1.76 | 1.13 | 0.96-1.34 | 1.36 | 1.07-1.73 |
|  | III | 1.47 | 1.26-1.73 | 1.57 | 1.26-1.96 | 1.50 | 1.29-1.75 | 1.69 | 1.37-2.09 |
|  | IV | 1.66 | 1.18-2.34 | 1.79 | 1.23-2.62 | 1.89 | 1.36-2.63 | 2.20 | 1.53-3.15 |
|  | Unknown | 1.43 | 1.11-1.85 | 1.51 | 1.10-2.08 | 1.55 | 1.21-1.98 | 1.77 | 1.30-2.40 |
| Operation | Abdominoperineal excision | 1.00 |  | 1.00 |  | 1.00 |  | 1.00 |  |
|  | Anterior resection | 0.67 | 0.57-0.79 | 0.54 | 0.44-0.67 | 0.67 | 0.57-0.78 | 0.55 | 0.45-0.68 |
|  | Hartmann’s procedure | 0.85 | 0.62-1.17 | 0.95 | 0.65-1.41 | 0.95 | 0.70-1.29 | 0.99 | 0.68-1.44 |
|  | Other | 1.35 | 1.11-1.63 | 1.47 | 1.19-1.82 | 1.44 | 1.20-1.73 | 1.51 | 1.23-1.86 |
